# Hypoxia-triggered degradable porphyrinic covalent organic framework for synergetic photodynamic and photothermal therapy of cancer

**DOI:** 10.1016/j.mtbio.2024.100981

**Published:** 2024-01-28

**Authors:** Yulong Liu, Kang Yang, Jun Wang, Yanzhang Tian, Bin Song, Ruiping Zhang

**Affiliations:** a General Surgery Department, Third Hospital of Shanxi Medical University, Shanxi Bethune Hospital, Shanxi Academy of Medical Sciences, Tongji Shanxi Hospital, Taiyuan, 030032, China; b Shanxi Medical University, Taiyuan, 030001, China; c The Radiology Department of Shanxi Provincial People’ Hospital, Five Hospital of Shanxi Medical University, Taiyuan, 030001, China

**Keywords:** Covalent organic framework, Hypoxia triggered, Degradable, Photoacoustic imaging, Photodynamic/photothermal synergistic therapy

## Abstract

Nanomedicines receive great attention in cancer treatment. Nevertheless, nonbiodegradable and long-term retention still limit their clinical translation. Herein, we successfully synthesize a hypoxia-triggered degradable porphyrinic covalent organic framework (HPCOF) for antitumor therapy *in vivo*. HPCOF possesses wide absorption in near infrared region (NIR) which endows HPCOF excellent photothermal conversion efficiency and photoacoustic (PA) imaging ability. Moreover, HPCOF exhibits excellent photodynamic and photothermal effect under special-wavelength laser irradiation. For the first time, the *in vitro* and *in vivo* tests demonstrate that HPCOF shows effective therapeutic effect for the combination of PDT and PTT under the monitoring of PA imaging. Importantly, in tumor region, HPCOF could be triggered by hypoxia microenvironment and collapsed gradually, then cleared from the body after treatment. This work fabricates a novel COF for cancer treatment and testifies great potential of HPCOF in clinical application with reducing long-term toxicity.

## Graphical abstract

**Figure:**
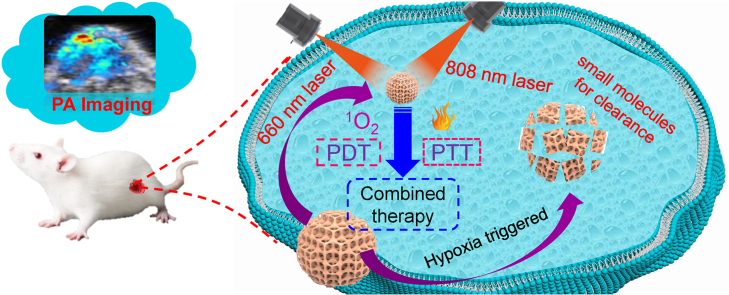

## 1 Introduction

**Scheme 1:**
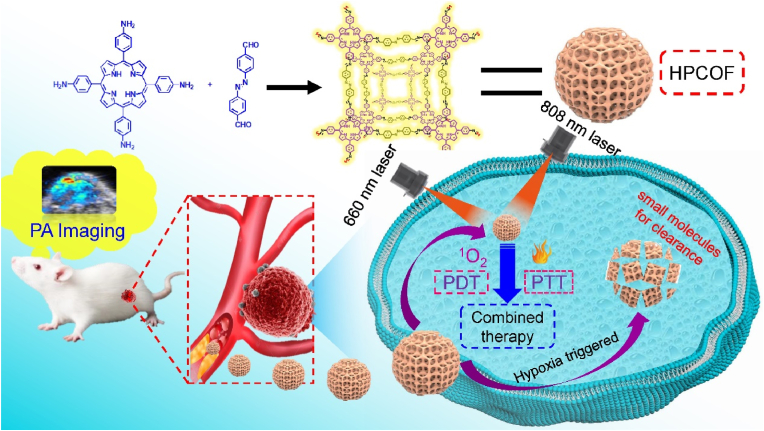
Schematic illustration of the preparation of HPCOF and photoacoustic imaging-guided combined therapy under 660 nm and 808 nm laser irradiation.

Nanotechnology holds tremendous potential in the diagnosis and treatment of cancer [1,2]. However, the uncontrollable and difficult biodegradability of the nanoparticles and long-time *in vivo* retention still cause severe and unpredictable toxicity risks [3]. Meanwhile, the poor biodegradability of the nanoparticles may prevent their clearance in the body [4,5]. The ideal nanomedicine should have a large size to enhance blood circulation time and tumor accumulation, and could be biodegraded under microenvironment stimuli in cancer treatment [6]. As one of the major pathological feature present in the microenvironment of most solid tumors, hypoxia is the result of the imbalance between oxygen intake and depletion, which is caused by abnormal blood vessels of tumor and the fast proliferation of cancer cells [[7], [8], [9], [10]]. Recently, introducing hypoxia-responsive motifs into the nano structure has been a new strategy for design and development of tumor microenvironment-triggered multifunctional therapeutic nanoplatforms [11]. Therefore, developing nanomedicines that are response to hypoxia microenvironment will bring new opportunities for cancer treatment (see Scheme 1).

Covalent organic frameworks (COFs) are built from organic building blocks via covalent bonds that crystallize into long-range-ordered two-dimensional (2D) or three-dimensional (3D) polymeric networks [12,13]. As an emerging class of porous crystalline polymers, COFs possess tremendous potential in many fields. Since the pioneering work was reported by Yaghi et al., COFs have attracted great attention for their tunable nature and porous characteristic [14]. To date, COFs have been used as promising platforms in sensor [15,16], catalysis [17], separation [18], and energy storage [19,20], etc. Moreover, COFs also were used for biomedical applications due to their enormous porosity and excellent structure stability, such as, drug delivery [[21], [22], [23]]. Importantly, some potential biological toxicity which caused by heavy metals in traditional inorganic nanomaterials can be avoided for their metal-free nature. However, the biomedical applications of the COFs also were limited by their large size which cause poor dispersibility and unsatisfactory bioavailability, so nanoscale size of COFs would make it suitable for biomedical purposes and overcome these obstacles [[24], [25], [26]].

In recent years, photodynamic therapy (PDT) has gained increasing attention and became a promising approach for cancer treatment due to its spatiotemporal selectivity and noninvasive nature. Under specific-wavelength light irradiation, photosensitizer (PS) transfer energy to O_2_ and produce ROS to damage target cells or tissue [27,28]. Similar to PDT, photothermal therapy (PTT) utilizes photothermal agents to convert light energy into heat and generate hyperthermia, then ablate various kinds of solid tumor by kill cancer cells [[29], [30], [31]]. Both PDT and PTT show great potential in cancer treatment and present small toxic and side effects when compared with traditional treatment. However, whether PDT or PTT, the single therapeutic strategy exhibits limited efficiency. Therefore, to achieve ideal therapy efficacy, the combination of PDT with other therapy strategies such as photothermal therapy (PTT), chemotherapy, and so forth has been realized recently.

Porphyrin-based derivatives exhibit good biocompatibility and show great potential for clinical translation. Thus, porphyrin-based COF could serve as promising candidates for biomedical application. Until now, many nano-size porphyrin-based COFs have been prepared and utilized in drug delivery [32], diagnosis [25], sonodynamic therapy (SDT) [33,34], PDT [[35], [36], [37]], PTT [38,39], and immunotherapy [[40], [41], [42]]. Nevertheless, Hypoxia-triggered and biodegradable porphyrin-based COFs have been little reported, and the unique performance has not been fully developed. Therefore, it is essential to developed hypoxia-triggered and biodegradable porphyrin-based COFs for biomedical applications and clinical translations, which could achieve excellent therapeutic efficacy with reduced toxicity.

In hypoxia physiological microenvironment, azobenzene group (AZO) can be reduced by azo reductase which is overexpressed in tumor tissues. Once the nanomedicines with AZO were taken up by cancer cells, the AZO can be reduced and cleaved by intracellular azo reductase, enabling triggered degradation of the nanomedicines into fragments for rapid clearance in body [43,44]. Herein, a nanoscale porphyrin-based hypoxia-triggered covalent organic framework, which named HPCOF, was synthesized by incorporating AZO into COF structure for the first time. The HPCOF showed good stability and could be stored in PBS for one week. Importantly, the prepared HPCOF was used to achieve photoacoustic (PA) imaging-guided PDT/PTT therapy *in vivo*. HPCOF possessed several novel and unique advantages: (1) HPCOF could prevent the quenching of porphyrin due to the increasing of the distance between molecules. (2) Compared to the porphyrin monomer, HPCOF had strong and broad absorption. Hence, HPCOF could be used as both photosensitizer and photothermal agent, produce ROS and heat under special wavelength light irradiation. (3) HPCOF was response to hypoxia microenvironment, and the AZO could be reduced and cleaved by intracellular azo reductase, enabling triggered degradation of the HPCOF into small molecules for rapid clearance. (4) The PA imaging ability of HPCOF would make it achieve precise treatment *in vivo*. (5) HPCOF is expected to be a promising candidate for drug delivery in cancer treatment due to its large surface areas and porous structure. In this work, our group synthetized porphyrin-based hypoxia-responsive covalent organic framework with nanoscale size for the first time and utilized it to achieve combined PDT and PTT therapy *in vivo*. This work not only provided a new structured nanomedicine with both PDT and PTT effects for cancer treatment, but also developed a new nanodrug delivery platform for accurate diagnosis and treatment of diseases.

## 2 Materials and methods

1, 3-diphenylisobenzofuran (DPBF) was purchased from Alfa Aesar (Tianjin, China). 2,2,6,6-tetramethylpiperidine (TEMP) was purchased from Meryer (Shanghai, China). Cell Counting Kit-8 was purchased from Changchun Sanbang Pharmaceutical Technology Co., Ltd. (Changchun, China). DCFH-DA and Calcein-AM/PI double stain kit were purchased from Beijing Solarbio Science & Technology Co., Ltd. (Beijing, China). IR820 was purchased from Shanghai Macklin Biochemical Technology Co., Ltd. (Shanghai, China). All of the chemicals and solvents were used as received without further purification.

### 2.1 Preparation of HPCOF

5,10,15,20-Tetrakis(4-aminophenyl)porphyrin (TAPP) and 4, 4'-Azodiphenylaldehyde were synthesized according to reported work [45,46]. TAPP (6.74 mg, 0.01 mmol) and 4,4'-Azodiphenylaldehyde (4.76 mg, 0.02 mmol) were mixed in 9 mL CH_2_Cl_2_. Then, 200 μL hydrofluoric acid was added. The mixture was stirred in the dark for 24 h. The precipitated black solids were collected by centrifugation (10000 rpm, 8 min). For further purification, the product was eluted with CH_2_Cl_2_ and water. The morphology and size of the HPCOF was characterized by TEM (JEM, F200) and DLS (malvern, zetasizer zs90).

### 2.2 Degradation of HPCOF

The reduction of HPCOF was carried out in PBS (10 mM, pH 7.4) at 37 °C for 6 h with the sodium dithionite (2 mM) which was employed to mimic azo reductase. TEM and DLS were also employed to investigate the morphology and hydrodynamic size of the HPCOF before and after reduction.

### 2.3 Photodynamic properties of HPCOF

1, 3-diphenylisobenzofuran (DPBF) was employed to investigate the ROS production. 20 μL of DPBF (1 mg/mL) was added to 3 mL of HPCOF aqueous solution (50 μg/mL). Then, the mixture was irradiated by a 660 nm laser with 450 mW/cm^2^. The absorption of DPBF was recorded at the predetermined time to detect the ROS production. And the DPBF + Laser group was set as control group to detect ROS production.

### 2.4 Photothermal properties of HPCOF

Various concentrations of HPCOF aqueous solution (100 μL) were added into EP tube and irradiated by an 808 nm laser (1.0 W/cm^2^) for 5 min, then the temperature change was recorded every 10 s by using a thermal imager. Simultaneously, the temperatures of the solutions (200 μg/mL) were also measured upon exposure to 808 nm laser under different power. The photothermal stability of HPCOF was evaluated by five on/off cycles under 808 nm laser irradiation (1.0 W/cm^2^). The photothermal conversion efficiency of HPCOF was calculated by following the reported method [47,48].

### 2.5 Cell culture

All the cells were incubated in DMEM medium with 10 % FBS and 1 % antibiotics (penicillin-streptomycin, 10000 U/mL) at 37 °C in a humidified atmosphere containing 5 % CO_2_. The 4T1 cell line was acquired from the Type Culture Collection of the Chinese Academy of Sciences (Shanghai, China).

### 2.6 Cellular uptake

4T1 cells were respectively seeded in confocal dishes and cultured for 24 h. After that, the 4T1 cells were washed with PBS and incubated with Cy5 labeled HPCOF in DMEM at 37 °C for 1, 2, 4, 6 and 8 h. Then, the cells were stained with DAPI for 10 min. Finally, the medium was removed and the cells were washed three times with PBS. The results were analyzed by CLSM (Olympus, FV3000).

### 2.7 Intracellular ROS generation

2’, 7’-dichlorofluorescein diacetate (DCFH-DA) was employed as the indicator to investigate the intracellular ROS generation. Briefly, 4T1 cells (1 × 10^4^) were seeded in a 96-well plate and cultured for 24 h. Then the HPCOF solution (200 μg/mL) was added. 6 h later, the cells were washed with PBS for three times and stained with DCFH-DA for another 30 min. After that, the cells were washed by PBS for three times to remove the excessive DCFH-DA. Finally, the cells were irradiated by a 660 nm laser for 5 min. Intracellular ROS generation were observed by using a fluorescence microscope.

### 2.8 Cell compatibility

4T1 and HeLa cells were seeded in the 96-well plate and cultured for 24 h. Then, different concentration (0, 6.25, 12.5, 25, 50, 100, 200, 400 μg/mL) of HPCOF DMEM solution were added into 96-well plate. 24 h later, CCK-8 assay was employed to measure the cells viability.

### 2.9 *In vitro* photocytotoxicity

To investigate the photocytotoxicity, four groups of 4T1 cells including HPCOF, HPCOF + 660 nm (0.5 W/cm^2^, 8 min), HPCOF + 808 nm (1 W/cm^2^, 5 min), HPCOF + 660 nm (0.5 W/cm^2^, 8 min) + 808 nm (1 W/cm^2^, 5 min) were seeded into 96-well plates and cultured for 24 h. Then, the original medium was removed and different concentration (6.25, 12.5, 25, 50, 100 μg/mL) of HPCOF was added, respectively. After incubation for 24 h, the cells were treated with the presumed methods. Then, the cells were incubated for another 4 h before the cell viability was detected by using the CCK-8 assay.

### 2.10 Live/dead cell staining assay

AM/PI assays has been used to evaluate the cytotoxicity of HPCOF by using fluorescence microscope observation. 4T1 cells were seeded in a 12-well plate and cultured for 24 h, then treated with PBS, HPCOF, HPCOF + 660 nm, HPCOF + 808 nm, and HPCOF + 660 nm + 808 nm. Four hours later, the cells were stained with the AM/PI for 30 min, and the excess dye was removed by washed with PBS. Finally, the cell images were obtained by using fluorescence microscope.

### 2.11 Animals and tumor model

All animal procedures were performed in accordance with the Guidelines for Institutional Animal Care and Use Committee, and approved by the Animal Ethics Committee of Shanxi Medical University (No. SYDL2019002). Female Balb/c mice (6–8 weeks old, 18–20 g) were obtained from Beijing Charles River Laboratory Animal Technology Co., Ltd. (China). To establish the tumor model, 4T1 cells (1 × 10^6^ cells, 50 μL) were subcutaneously injected 4T1 cells into the right thigh of each mice.

### 2.12 *In vivo* PA

For *in vivo* PA imaging, 4T1 tumor-bearing Balb/c mice were intravenously injected with HPCOF (1.0 mg/mL, 200 μL), followed by tumor imaging with the Visual sonic Vevo LAZR-X imaging system at time points of 0, 2, 4, 6, 8, 10, 12 and 24 h. The dynamic change trends of PA signals located at tumor sites were analyzed.

### 2.13 *In vivo* fluorescence imaging

The 4T1 tumor-bearing Balb/c mice were intravenously injected with IR820 labeled HPCOF for monitoring the biodistribution of HPCOF. After intravenous injection of IR820@HPCOF, same to the PA imaging *in vivo*, the fluorescence images were obtained at different time points on live animal imaging system (Series III 900/1700-D, Suzhou NIR-Optics Technologies Co., Ltd.).

### 2.14 *In vivo* combined PDT and PTT therapy

When the tumor volume of mice reached to about 100 mm^3^, all the mice were divided into 6 groups including PBS, HPCOF, 660 nm + 808 nm, HPCOF + 660 nm, HPCOF + 808 nm, HPCOF + 660 nm + 808 nm. Then the mice were intravenously injected with HPCOF (1 mg/mL, 200 μL) on day 1, day 3 and day 5. 12 h later, the treated groups of mice were irradiated with 660 nm (0.5 W/cm^2^, 8 min) and 808 nm (1 W/cm^2^, 5 min) laser. The tumor volume and body weight of all mice were recorded every two days in 14 d to evaluate the therapeutic effect *in vivo*. Tumor volume was obtained based on the equation: V(mm^3^) = length × (width)^2^/2.

After treatment, tumors and major organs of the mice (heart, liver, spleen, lungs, and kidneys) of each group of mice were collected for H&E staining. TUNEL staining assays were employed to evaluate the apoptosis of tumor cells. For long-time toxicity assessment, healthy female Balb/c mice were intravenously injected with HPCOF (10 mg kg^−1^) on days 1, 3, 7, and 14 prior to execution, and control mice were administered PBS. Blood samples were collected for blood routine and blood chemistry (liver and renal function) examination.

## 3 Results and discussion

### 3.1 Preparation and characterization of HPCOF

Inspired by the reported work, nanosized HPCOF was facilely fabricated by a solution-based aging method at room temperature [36]. Briefly, 5,10,15,20-Tetrakis(4-aminophenyl) porphyrin (TAPP) [45] and 4, 4'-Azodiphenylaldehyde (Apa) [46,49] were mixed in CH_2_Cl_2_ solution and stirred in the dark overnight. The dark product was isolated by centrifugation, then washed with CH_2_Cl_2_ and water.

**Fig. 1:**
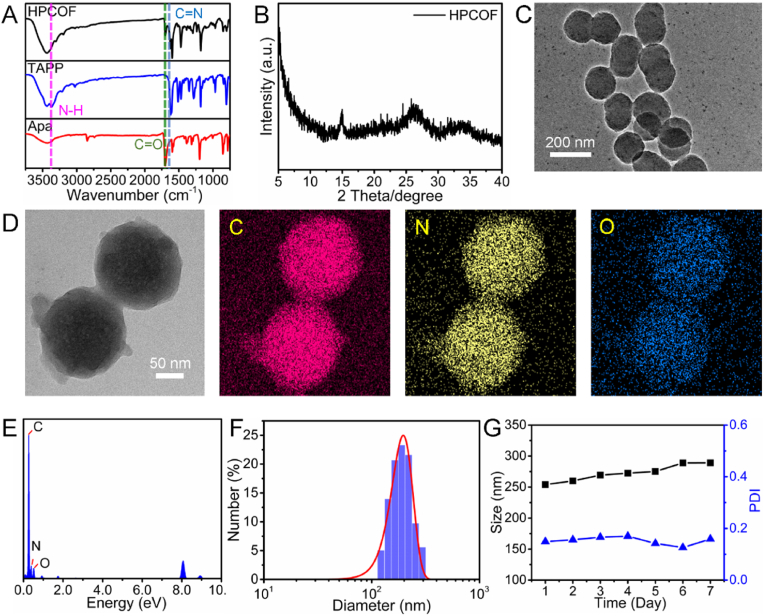
A) FT-IR spectra of HPCOF, TAPP and Apa. B) PXRD pattern of HPCOF. C) TEM image of HPCOF. D) Elemental mapping of C, N and O in HPCOF. E) EDS spectrum of HPCOF. F) Hydrodynamic size distribution of HPCOF. G) The hydrodynamic size and polydispersity index (PDI) changes of HPCOF in one week.

The successful synthesis of HPCOF was characterized by using Fourier transform infrared (FT-IR) spectroscopy and PXRD pattern. In FT-IR spectra (Fig. 1A), the characteristic peak of C=N vibration was observed at 1621 cm^−1^, and the relative intensity of stretching bands for C=O and N−H decreased respectively, which demonstrating the successful condensation between the TAPP and 4, 4'-Azodiphenylaldehyde. PXRD pattern of the HPCOF was conducted to investigate the crystal property. As shown in Fig. 1B, HPCOF exhibited one intensive peak at 14.9°, and two medium diffraction peaks at 26.2° and 33.7°. Compared with the highly crystalline porous materials, HPCOF showed amorphous crystallization for its relatively few characteristic peaks. The morphology and size of the HPCOF was characterized by transmission electron microscopy (TEM). As shown in Fig. 1C, HPCOF showed uniform spherical morphology with a particle size of about 140 nm. The TEM mapping images (Fig. 1D) and energy dispersive spectra (Fig. 1E) demonstrated that the homogeneous distribution of C, N, and O elements. The dynamic light scattering (DLS) measurements of the HPCOF were further conducted. As shown in Fig. 1F, the dynamic hydraulic diameter of HPCOF in water was about 200 nm which was larger than the result of TEM. The ζ-potential of the HPCOF was 30.8 mV (Fig. S1). The stability of the HPCOF was investigated in PBS. As shown in Fig. 1G, the size of the HPCOF was unchanged and stable even after storage for one week, suggesting a good stability of the HPCOF.

**Fig. 2:**
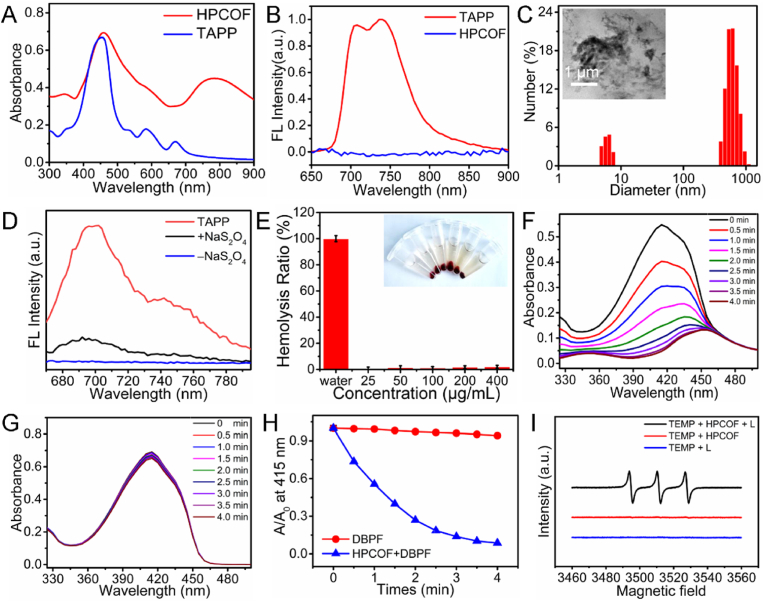
A) UV–vis absorption spectra of HPCOF and TAPP. B) Fluorescence spectra of HPCOF and TAPP in THF. C) Hydrodynamic size distribution and TEM image (inset) of HPCOF after treatment of Na_2_S_2_O_4_. D) Fluorescence spectra of HPCOF with and without Na_2_S_2_O_4_ in PBS. E) Hemolysis ratio and images of RBCs treated with HPCOF. Time-dependent UV–vis absorption spectra of DPBF F) with or G) without the presence of HPCOF in water after irradiation with 660 nm laser (0.5 W/cm^−2^) for different time (0–4 min). H) UV–vis absorption changes curves of DPBF at 415 nm under different treatments (with or without the presence of HPCOF). I) ESR spectra of TEMP + Laser, HPCOF + TEMP and HPCOF + TEMP + Laser.

Porphyrin has been widely used as photosensitizer for PDT in clinic. The photophysical properties of HPCOF were investigated. The UV–Vis spectra (Fig. 2A) of HPCOF showed red shifted when compared with the monomer, TAPP. As shown in Fig. 2A, TAPP showed Soret band at 450 nm and Q bands at 534, 581, and 670 nm; however, only two relatively wide absorption bands at 460 and 780 nm were observed in HPCOF. This phenomenon probably should be ascribed to the enhanced π-π interaction and the formation of the conjugation structure [33,50]. The fluorescence properties of HPCOF were further investigate in THF and PBS solution. As shown in Fig. 2B and Fig. S2, the porphyrin monomer, TAPP, exhibited stronger fluorescence signal when compared with HPCOF. Because of the energy conservation, HPCOF may have the potential to produce PDT or PTT effect under special-wavelength laser irradiation.

Biosafety is a main concern in clinical translation. Hence, sufficient biodegradability of the COF plays an important role in biomedical applications. The responsiveness of the HPCOF to hypoxia microenvironment *in vitro* has been evaluated. Here, sodium hydrosulfite (Na_2_S_2_O_4_) was chosen to biomimetic azo reductases and mixed completely with HPCOF in PBS at 37 °C, then the results was analyzed by DLS and TEM [44,51]. As shown in Fig. 2C, compared to the initial HPCOF, the original spherical structure of HPCOF was destroyed entirely, and the size distribution of the HPCOF changed from unimodal one to bimodal size distribution. Interestingly, the fluorescence signals of the porphyrin monomer appeared after adding Na_2_S_2_O_4_ to HPCOF solution (Fig. 2D). These results demonstrated that the HPCOF was triggered by biomimetic azo reductases Na_2_S_2_O_4_ and could be degraded under tumor hypoxia microenvironment.

### 3.2 Hemolysis assay

The hemocompatibility of HPCOF was tested to investigate the safety of the nanomedicine. As shown in Fig. 2E, when the concentration of HPCOF was up to 400 μg/mL, there was no obvious hemolysis was observed, indicating that HPCOF had excellent hemocompatibility.

### 3.3 Photodynamic properties of HPCOF

To investigate the photodynamic effect of HPCOF, 1,3-diphenylisobenzofuran (DPBF) was employed as a singlet oxygen (^1^O_2_) indicator to investigate the photodynamic performance. As shown in Fig. 2F–H, when HPCOF were under 660 nm laser irradiation, the UV–vis absorbance intensity of DPBF at 415 nm was decreased with increasing irradiation time. In sharp contrast, the control group exhibited no obvious change in absorbance at 415 nm under laser irradiation in the absence of HPCOF. In addition, singlet oxygen sensor green (SOSG) was also used to investigated the ROS (^1^O_2_) generation property of HPCOF. As shown in Fig. S3, the fluorescence intensity of SOSG was increased gradually when HPCOF under 660 nm laser irradiation. In contrast, the fluorescence intensity of SOSG in the control group was almost unchanged under 660 nm laser irradiation. These results indicated that HPCOF can generate ^1^O_2_ under laser irradiation with special-wavelength.

Furthermore, electron spin resonance (ESR) spectra were employed to verify the generation of ^1^O_2_ by using 2,2,6,6-tetramethylpiperidine (TEMP) as the ^1^O_2_ trapping agent. As shown in Fig. 2I, we found that the mixture of HPCOF and TEMP can produce typical 1:1:1 peak signal after 660 nm laser irradiation. In contrast, there was no obvious ESR signal was observed for TEMP + L and TEMP + HPCOF groups. These results indicated that HPCOF can produce ^1^O_2_ under 660 nm laser irradiation. The mechanism for the HPCOF could produce ^1^O_2_ may can be explained by the exist of the porphyrin in the structure, under specific-wavelength laser irradiation, HPCOF absorbs energy and was excited to triplet state, the instable excited HPCOF transfers the energy to O_2_ and produce ^1^O_2_ when back to ground state [[52], [53], [54]].

### 3.4 Photothermal properties of HPCOF

In Fig. 2A, the UV–vis absorption spectrum of HPCOF exhibited a broad absorption peak at 780 nm, which indicated HPCOF possessed enhanced absorbance at NIR region. Hence, we inferred that HPCOF have excellent photothermal property. The photothermal effect of HPCOF was systematically evaluated under 808 nm laser irradiation.

**Fig. 3:**
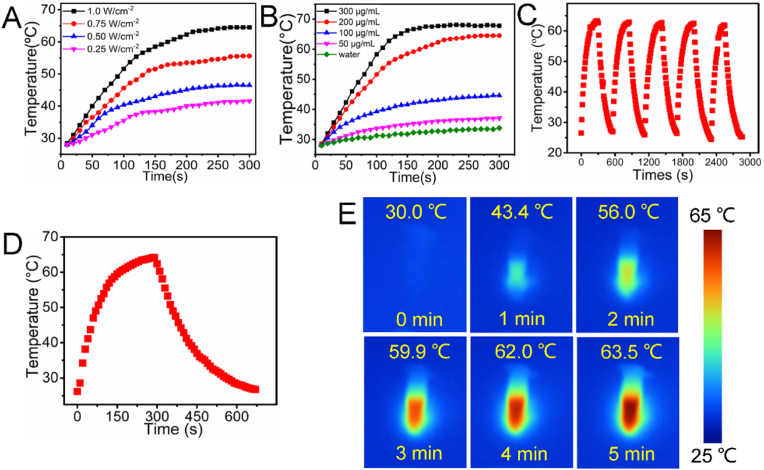
A) Photothermal heating curves HPCOF NPs with 200 μg/mL under 808 nm laser irradiation at different power density. B) Photothermal heating curves of HPCOF under 808 nm laser irradiation (1.0 W/cm^2^) at different concentrations. C) Temperature variations of HPCOF under 808 nm laser irradiation (1.0 W/cm^2^) over five cycles of heating/cooling. D) One cycle of heating and cooling of HPCOF for the calculation of photothermal conversion efficiency. E) Thermal images of HPCOF with 808 nm laser irradiation at 1 W/cm^2^ within 5 min.

As shown in Fig. 3A, the temperature of HPCOF solution increased quickly with the laser density and time increased. When the concentration of HPCOF was 200 μg/mL, the solution temperature increased from 28 °C to 64 °C in 5 min with laser density of 1.0 W/cm^2^. The temperature change of the HPCOF solution at different concentrations was also conducted under 808 nm laser irradiation with laser density of 1.0 W/cm^2^ and pure water was chosen as the control. As shown in Fig. 3B, the photothermal effect of HPCOF was closely related to the concentration. The temperature of HPCOF increased from 28 °C to 67 °C in 5 min (1.0 W/cm^2^) when the concentration was 300 μg/mL, in contrast, there was no obvious temperature increase for pure water. The photothermal stability of HPCOF was also evaluated under five consecutive laser on-off cycles. As shown in Fig. 3C, there was no obvious change was observed in the maximal temperature during each cycle, indicating the excellent photothermal stability of HPCOF. According to the reported method, the photothermal conversion efficiency of HPCOF was calculated to be ∼35 % (Fig. 3D and S3) [48]. The experiment of thermography imaging *in vitro* has been carried to visualize temperature change of HPCOF under 808 nm laser irradiation. As shown in Fig. 3E, the HPCOF solution exhibited a rapid temperature rise from 30 °C to 63.5 °C. These results indicated that HPCOF can be used as a PTT agent for cancer therapy.

### 3.5 Cell experiment of HPCOF

Encouraged by the potential phototherapy ability, we explored the antitumor performance of the HPCOF on cellular level. Firstly, the cellular uptake of HPCOF was evaluated in 4T1 cells at different times. Here, Cy5 was employed as fluorescent dye to label HPCOF for investigating the cellular uptake. And the cellular uptake of HPCOF against 4T1 cells was confirmed by using confocal laser scanning microscope (CLSM). As shown in Fig. S4 (Supporting Information), the red fluorescence of Cy5 in cytoplasm enhanced obviously with the lengthening of incubation time. This result indicated that HPCOF could be effectively endocytosed into the cells.

**Fig. 4:**
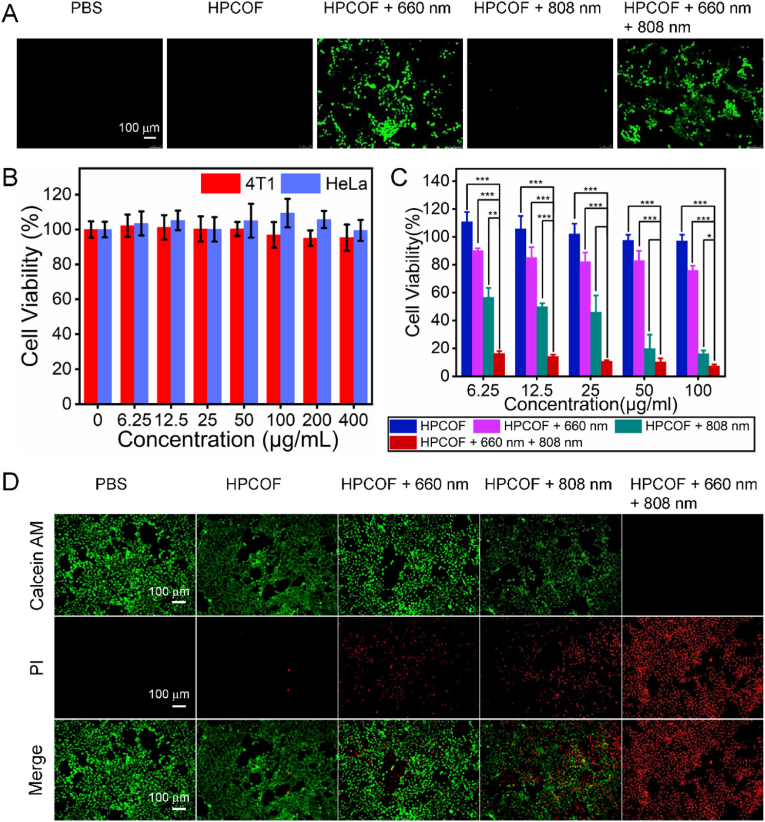
A) Intracellular detection of ROS production in 4T1 cells after different treatments with DCFH-DA as the probe. B) Viability of 4T1 and HeLa cells after treatment with different concentrations of HPCOF. C) Viability of 4T1 cells after various treatment. Statistical significance: ***p < 0.001, **p < 0.01, *p < 0.05. D) Fluorescent images of Calcein-AM/PI stained 4T1 cells after different treatments.

In order to investigate intracellular ROS production, 2′,7′-dichlorofluorescin diacetate (DCFH-DA) was employed as an indicator to evaluate the intracellular ROS production, characterized by bright green fluorescence in cells. DCFH-DA has no fluorescence when it spreads to the cells, once it is oxidized by ROS to DCF, can produce bright green fluorescence. As shown in Fig. 4A, the 4T1 cells treated with PBS, HPCOF and HPCOF + 808 nm laser irradiation showed negligible green fluorescence. In contrast, the cells treated with HPCOF + 660 nm laser and HPCOF + 660 nm + 808 nm laser irradiation exhibited obvious green fluorescence, which indicated that ROS was generated in these groups.

Biocompatibility and cell phototoxicity of HPCOF has also been assessed. HeLa and 4T1 cells were treated with various concentrations (0−400 μg/mL) of HPCOF for 24 h in the absence of irradiation, then the viability the cells was evaluated by using CCK-8. As shown in Fig. 4B, the cell viability of HeLa and 4T1 cells was over 90 % even if the concentration of HPCOF was up to 400 μg/mL, indicating HPCOF possess low cytotoxicity and excellent biocompatibility. In contrast, the cell viability decreased rapidly under 660 nm or 808 nm laser irradiation in the presence of HPCOF, especially for the HPCOF + 660 nm + 808 nm group. These results indicated effective antitumor effect of the combination of PDT and PTT. In addition, the photocytotoxicity of HPCOF were highly dependent on the concentration. Under the same conditions, HPCOF showed better photocytotoxicity with the increasing concentration (Fig. 4C).

Calcein-AM and PI double staining was conducted to confirm the cell cytotoxicity of HPCOF *in vitro*. As shown in Fig. 4D, PBS and only HPCOF groups presented strong green signal, indicating negligible cell death. In contrast, HPCOF + 660 nm and HPCOF + 808 nm groups presented obvious red signal, indicating the cells were almost death. The HPCOF + 660 nm + 808 nm group exhibited strongest red signal, indicating that HPCOF could kill more cancer cells by combined PDT and PTT. These results were also in agreement with the CCK8 assay.

### 3.6 *In vivo* biodistribution and photothermal measurements

**Fig. 5:**
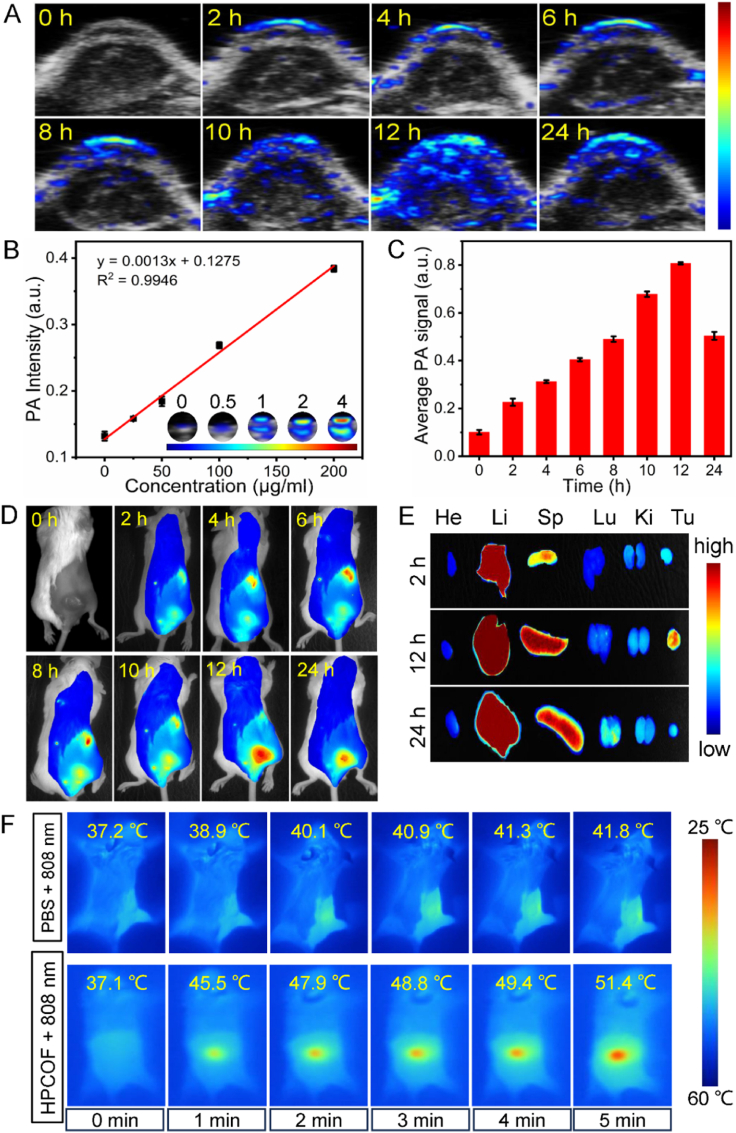
A) Photoacoustic (PA) images of mice after intravenous injection of HPCOF at different time points. B) PA imaging of HPCOF solutions with different concentrations. C) Intensities of PA signals in tumor at different times. D) Fluorescence images of mice after intravenous injection of HPCOF at different time points. E) Fluorescent images of tumor tissues and major organs after intravenous injection of HPCOF at 2 h, 12 h and 24 h. F) Thermal images of tumor-bearing mice injected with PBS or HPCOF under 808 nm laser (1.0 W/cm^2^) irradiation for 5 min.

As a noninvasive imaging technique, PA imaging has attracted great attention for its deep penetration and high sensitivity properties [[55], [56], [57]]. Due to the broad absorption in NIR region, much stronger PA signal was observed along with the increasing concentration of HPCOF (Fig. 5B). The biodistribution of HPCOF was further examined by using an *in vivo* PA imaging system (Visual sonic Vevo LAZR-X imaging system), which can provide a suitable time point for treatment. And 4T1 tumor-bearing Balb/c mice was used as the experimental model. Then, the mice were intravenously injected with HPCOF, and PA signals were monitored at different time points. As shown in Fig. 5A and C, the PA signals in the tumor site increased gradually to reach a maximum after 12 h of intravenous injection, indicating the accumulation of HPCOF in tumor site driven by EPR effect. Thus, 12 h was chosen as the suitable time point for phototherapy. As time goes on, the PA signals decayed gradually in the tumor site. Therefore, the PA imaging-guided treatment could be achieved. Furthermore, longer time was conducted to monitor the PA signals for investigating the time of being expelled from the body. As shown in Fig. 5A and S6, the PA signals were decreased gradually as time goes on, and there was almost no PA signals can be observed 5 days later (Fig. S6). These results suggested that HPCOF can be expelled from the body over time.

Similar experiments were also conducted by using fluorescence imaging. And a NIR dye, new indocyanine green (IR820), was employed to label the HPCOF for fluorescence imaging. As shown in Fig. 5D, HPCOF could accumulate effectively at tumor site and it reached a maximum at 12 h after intravenous injection, which in agreement with the PA imaging result. In order to further investigate the biodistribution of HPCOF, the tumor and main organs were extracted from euthanized mice at different postinjection time points for *ex vivo* NIR fluorescence imaging. As shown in Fig. 5E, the tumor tissue exhibited distinct fluorescence signals despite obvious signals were also found in the metabolic organ of liver. Therefore, the results of the PA imaging and fluorescence imaging provided a suitable time point for treatment.

Furthermore, the degradation of HPCOF in the hypoxia and tumor microenvironment has also been investigated by PA imaging monitor *in vivo*. Herein, HPCOF solution was locally injected into the muscle and tumor, respectively, then monitored with PA imaging at different time points. As shown in Fig. S7, the PA signals of the muscle was almost unchanged from 2 to 10 h postinjection, however, the PA signals of the tumor was significantly reduced over time. These results indicated that HPCOF was response to hypoxia and tumor microenvironment, and could be degraded.

The PTT effect of HPCOF also has been evaluated on 4T1 tumor-bearing mice, and thermal imaging was employed to monitor the temperature changes in the tumor area. After intravenous injection of HPCOF for 12 h, the tumor area was exposed to 808 nm laser for 5 min and the temperature changes was monitored. As shown in Fig. 5F, in the HPCOF + 808 nm group, the temperature of the tumor area raised quickly from 37.1 to 51.4 °C in 5 min, demonstrating the accumulation of HPCOF and excellent photothermal conversion achieved by HPCOF. However, For the mice treated with PBS + 808 nm, the temperature only changed from 37.2 to 41.8 °C under the same condition. These results further confirmed the excellent photothermal effect of HPCOF.

### 3.7 *In vivo* phototherapy

**Fig. 6:**
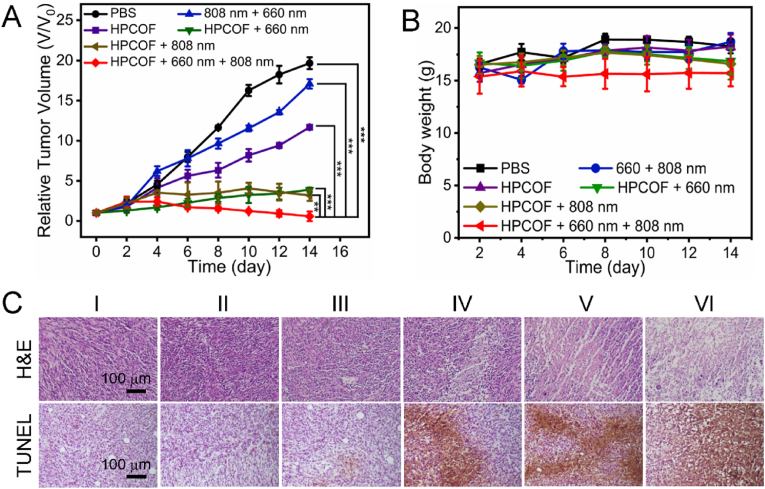
A) Relative tumor volume after various treatments. Statistical significance: ***p < 0.001, **p < 0.01. B) Body weight of mice during various treatments. C) H&E and TUNEL staining of tumor tissues of mice after various treatments for 14 days. (I) PBS, (II) HPCOF, (III) 660 nm + 808 nm, (IV) HPCOF + 660 nm, (V) HPCOF + 808 nm, (VI) HPCOF + 660 nm + 808 nm.

Inspired by the outstanding performance and good tumor accumulation of HPCOF, we conducted the therapeutic evaluation *in vivo*. 4T1 tumor-bearing Balb/c mice model was employed to investigate the therapeutic effect of HPCOF. When the tumor volumes reached ∼100 mm^3^, the mice were divided into six groups: PBS, HPCOF, 660 nm + 808 nm, HPCOF + 660 nm, HPCOF + 808 nm and HPCOF + 660 nm + 808 nm. After intravenous injection of HPCOF for 12 h, the tumor area was subjected to laser irradiation (660 nm, 0.5 W/cm^2^, 8 min and 808 nm, 1 W/cm^2^, 5 min). Then, the tumor volume and body weights of the mice were measured every 2 days in the following 14 days. As shown in Fig. 6A, HPCOF and 660 nm + 808 nm groups had little effect on tumor suppression. Both the PDT and PTT effects of HPCOF showed an inhibitory effect on the tumor. Significantly, tumor growth can be completely inhibited and ablated after being treated with HPCOF + 660 nm + 808 nm, demonstrating the excellent therapeutic effect of the combined therapy (Fig. S9). We further evaluated the body weight change of the mice during treatments. In these six groups, the body weight of mice showed no obvious changes, indicating the low toxicity of HPCOF (Fig. 6B).

**Fig. 7:**
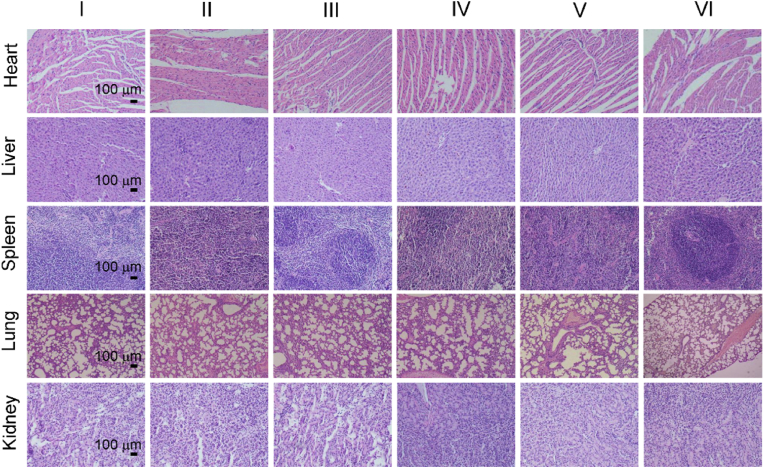
H&E images of major organs at the end of various treatments. (I) PBS, (II) HPCOF, (III) 660 nm + 808 nm, (IV) HPCOF + 660 nm, (V) HPCOF + 808 nm, (VI) HPCOF + 660 nm + 808 nm.

After treatments, major organs and tumor tissues were collected and sliced for histological analysis. As shown in Fig. 6C, H&E stained and TUNEL stained results of tumors showed the combination of PDT and PTT could effectively damage tumor tissue. H&E staining of the major organs of the mice show no obvious pathological damage (Fig. 7). Blood routine and blood biochemistry also were conducted to investigated the long-term toxicity of HPCOF *in vivo* (Fig. S8). Healthy female Balb/c mice were intravenously injected with 10 mg kg^−1^ of HPCOF on day 1, 3, 7, and 14 prior to execution, and the control group mice were intravenously injected with PBS. Blood routine indices were found to be within the normal reference range according to routine blood tests. And blood biochemical results showed that HPCOF had no significant effects on liver and kidney function. These results confirmed that HPCOF achieved anticancer performance under laser irradiation via the combination of PTT and PDT, and showed highly biocompatible.

## 4 Conclusion

In summary, we developed a novel covalent organic framework with excellent PDT and PTT efficiency. Under special-wavelength laser irradiation, HPCOF can produce ^1^O_2_ and heat, which can be used for synergetic PDT and PTT therapy. Subsequently, the combined therapeutic effect of HPCOF was verified both *in vitro* and *in vivo*. Moreover, we used HPCOF to achieve PA imaging-guided synergetic PDT and PTT cancer treatment *in vivo* for the first time. Importantly, in tumor microenvironment, HPCOF could be triggered by hypoxia and degraded into small molecules for clearance after treatment, which exhibited good biosafety. We expect that this work not only provides a new structure COF with good biosafety for cancer treatment, but also builds a new drug delivery system for accurate diagnosis and treatment of diseases.

## CRediT authorship contribution statement

**Yulong Liu:** Writing – original draft, Project administration, Investigation, Data curation, Conceptualization. **Kang Yang:** Methodology, Formal analysis, Data curation. **Jun Wang:** Visualization, Methodology. **Yanzhang Tian:** Visualization, Methodology. **Bin Song:** Visualization, Methodology. **Ruiping Zhang:** Writing – review & editing, Supervision, Conceptualization.

## Declaration of competing interest

The authors declare that they have no known competing financial interests or personal relationships that could have appeared to influence the work reported in this paper.

## Data Availability

Data will be made available on request.
