# Supplementary material for: Hypoxia-triggered degradable porphyrinic covalent organic framework for synergetic photodynamic and photothermal therapy of cancer
Source: Mater Today Bio. 2024 Jan 28;25:100981. doi: 10.1016/j.mtbio.2024.100981 (PMC10865025; doi:10.1016/j.mtbio.2024.100981)
Supplement: Multimedia component 1 [file mmc1.docx]

**Hypoxia-Triggered Degradable Porphyrinic Covalent Organic Framework for Synergetic Photodynamic and Photothermal Therapy of Cancer**

Yulong Liu^a,b,1^, Kang Yang^b,1^, Jun Wang^a^, Yanzhang Tian^a^, Bin Song^a^, and Ruiping Zhang^c,^^*^

**Affiliations:**

^a^General Surgery Department, Third Hospital of Shanxi Medical University, Shanxi Bethune Hospital, Shanxi Academy of Medical Sciences, Tongji Shanxi Hospital, Taiyuan 030032, China

^b^Shanxi Medical University, Taiyuan 030032, China

^c^The Radiology Department of Shanxi Provincial People’ Hospital, Five Hospital of Shanxi Medical University, Taiyuan 030001, China

*Corresponding author at: The Radiology Department of Shanxi Provincial People’ Hospital, Five Hospital of Shanxi Medical University, Taiyuan 030001, China

E-mail: [zrp_7142@sxmu.edu.cn](mailto:zrp_7142@sxmu.edu.cn) (R. Zhang)

^1^These authors contribute equally to this work


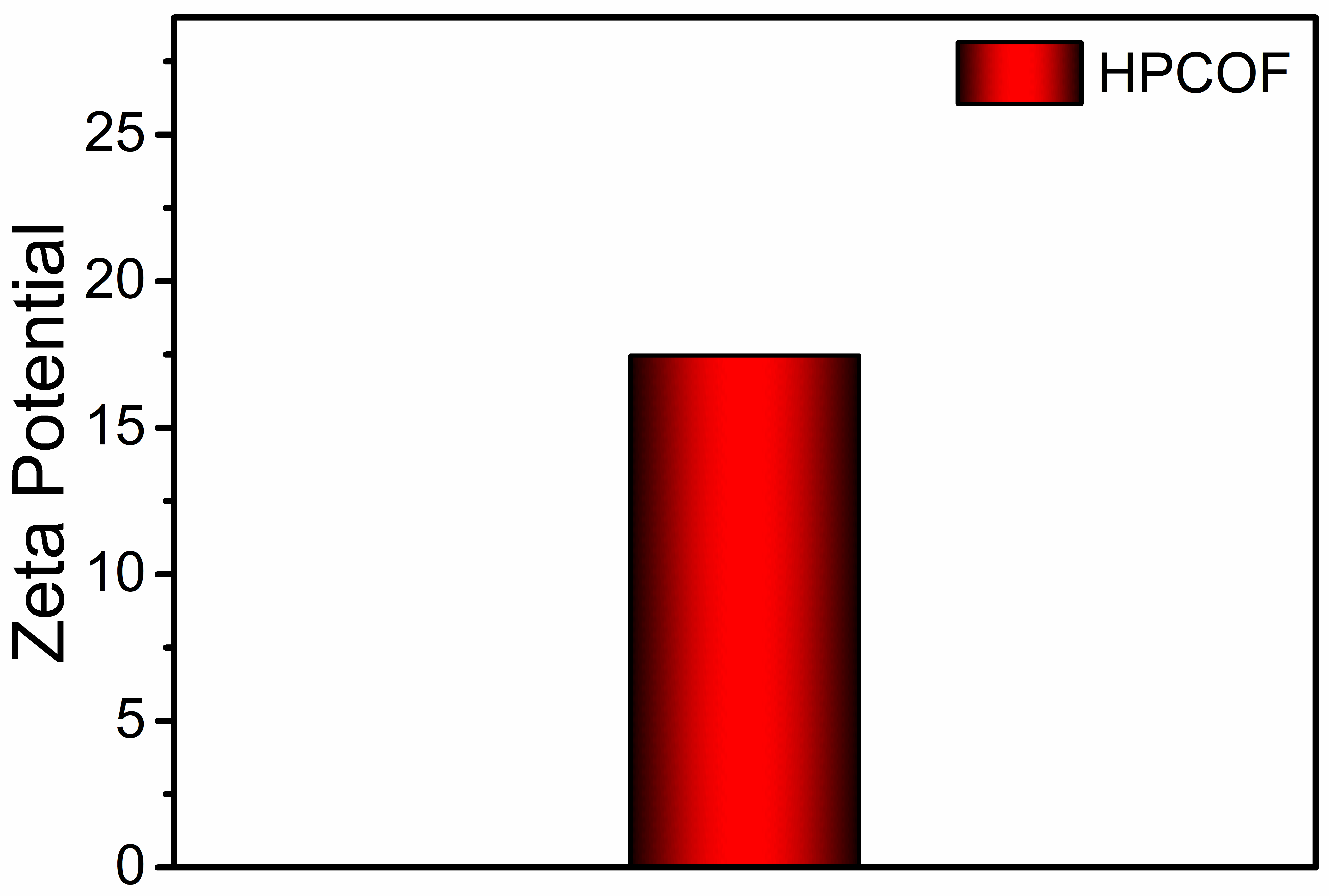


**Figure S1.** Zeta potential of HPCOF.


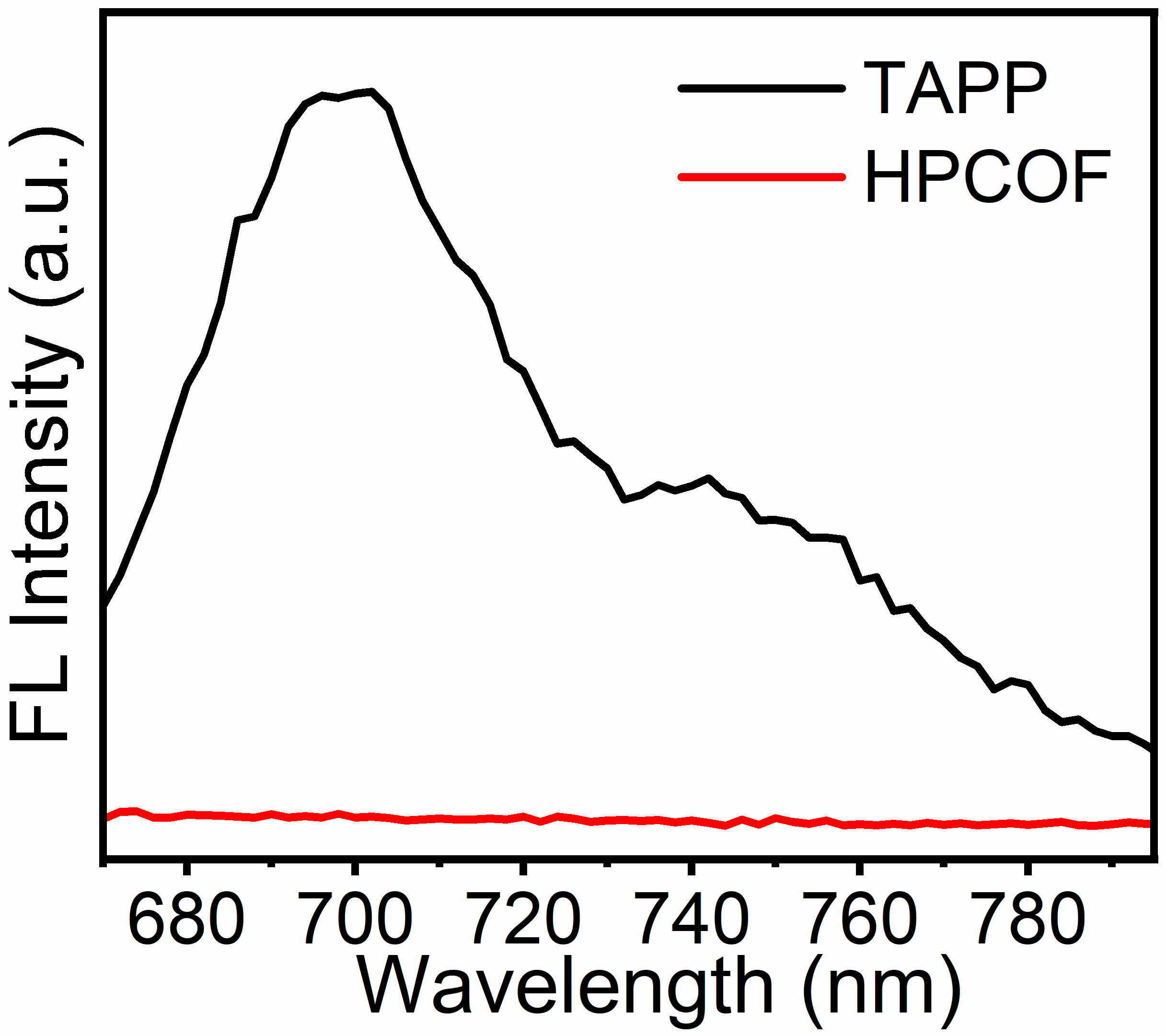


**Figure S2.** Fluorescence spectra of HPCOF and TAPP in PBS.


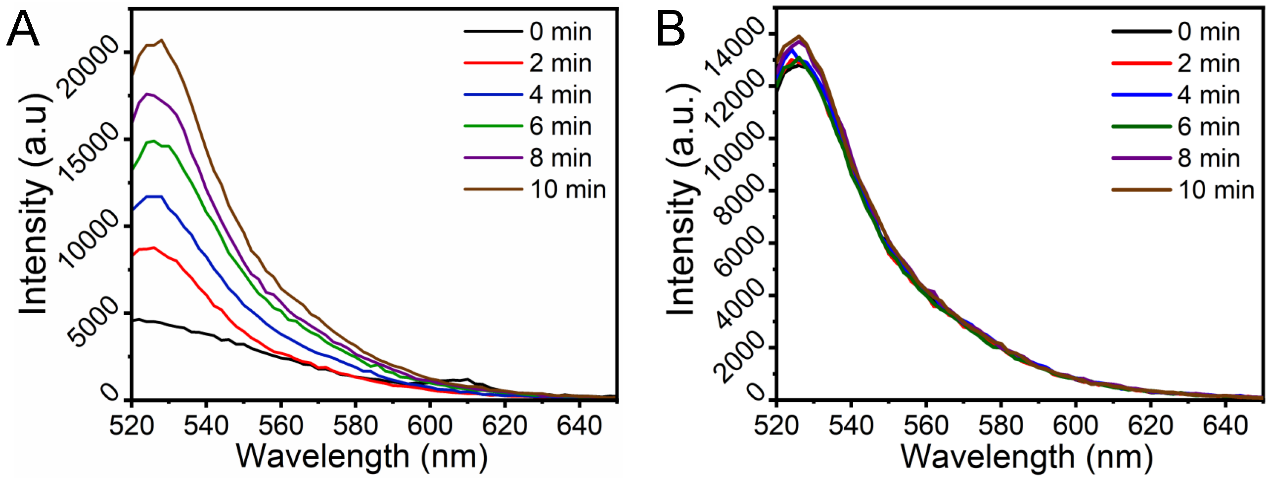


**Figure S3.** Time-dependent fluorescence intensity of SOSG A) with or B) without the presence of HPCOF after irradiation with 660 nm laser for different time.


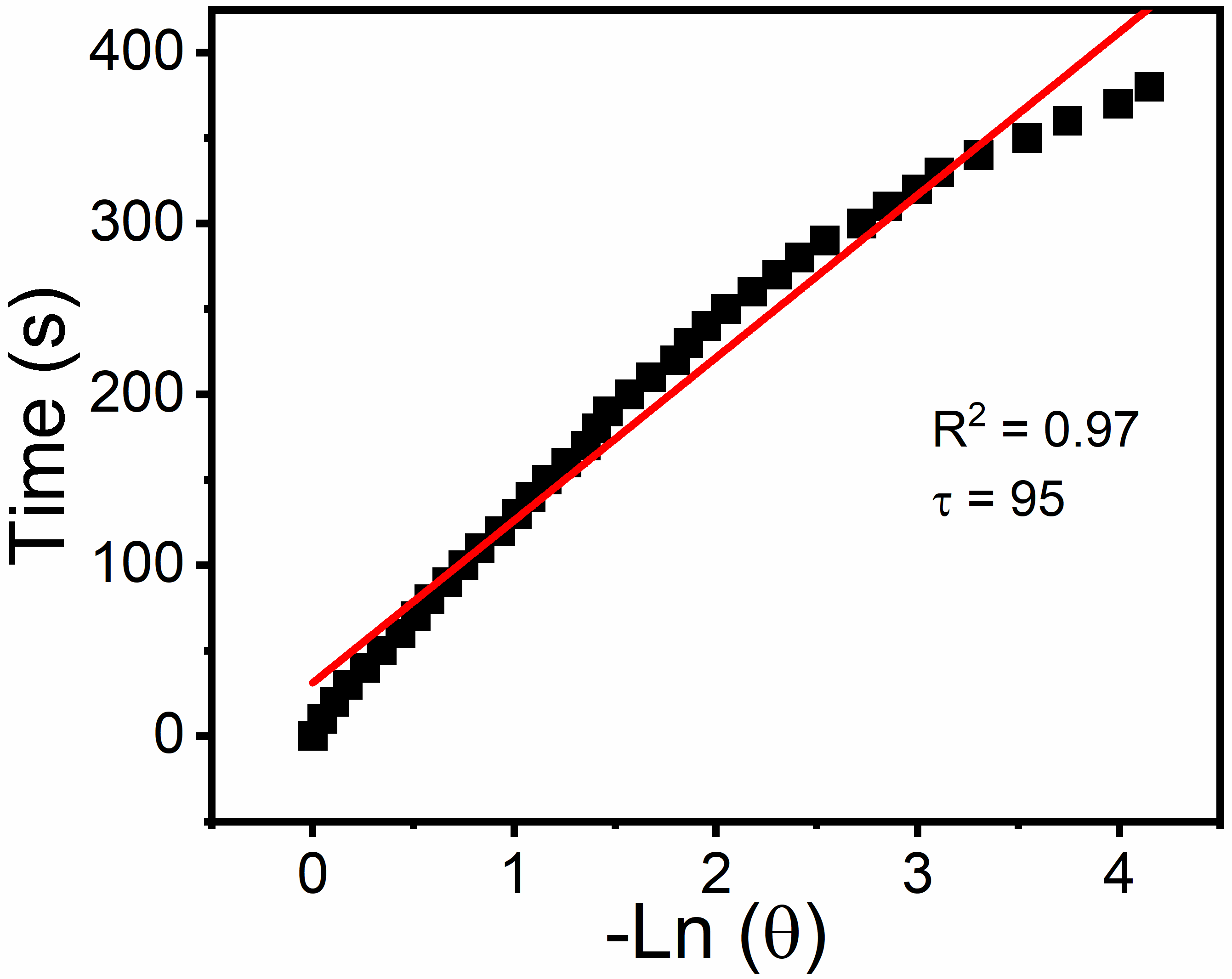


**Figure S4.** Linear fit of time/-ln(θ) obtained during the cooling process.


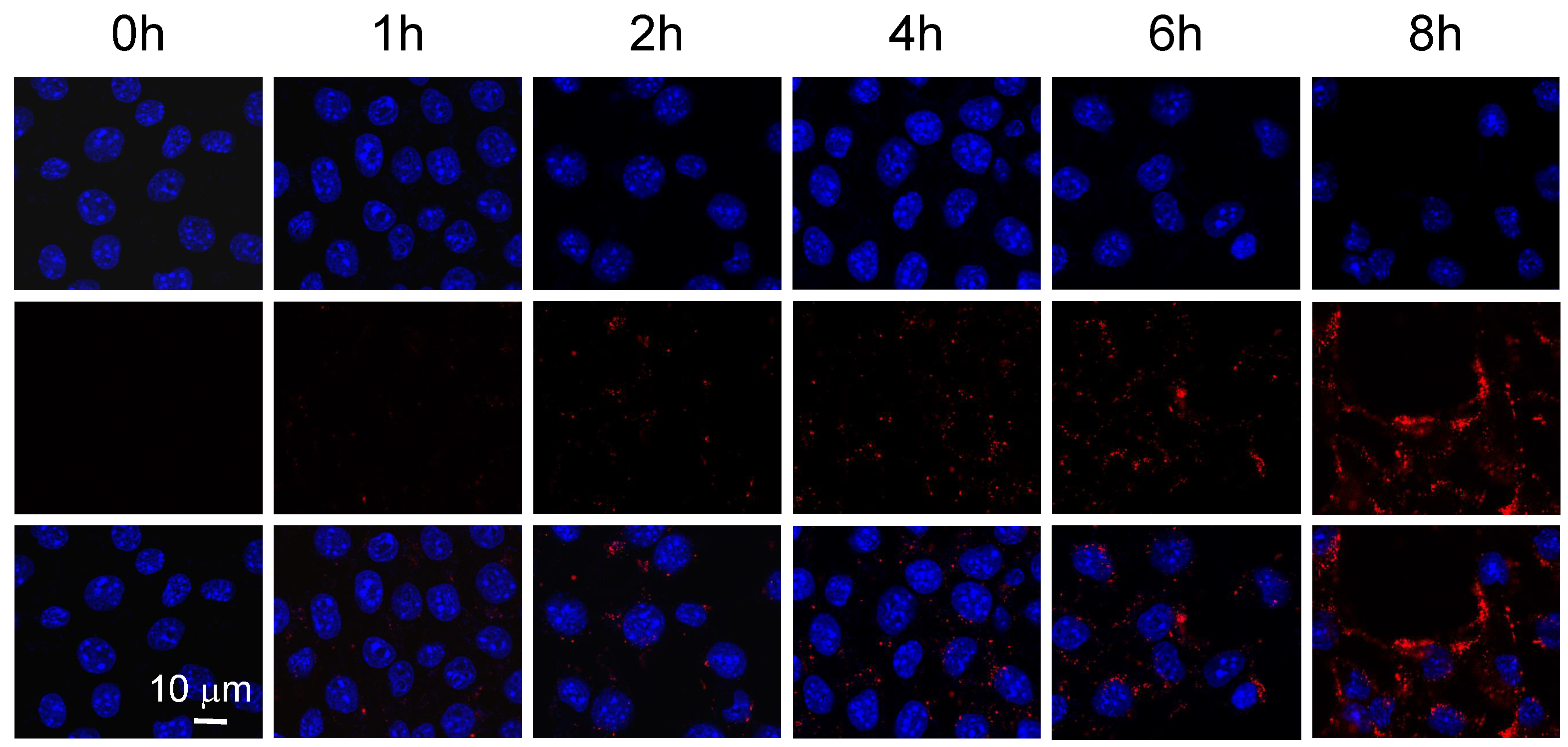


**Figure S5.** Cy5 labeled HPCOF Laser confocal images of 4T1 cells cultured at different times.


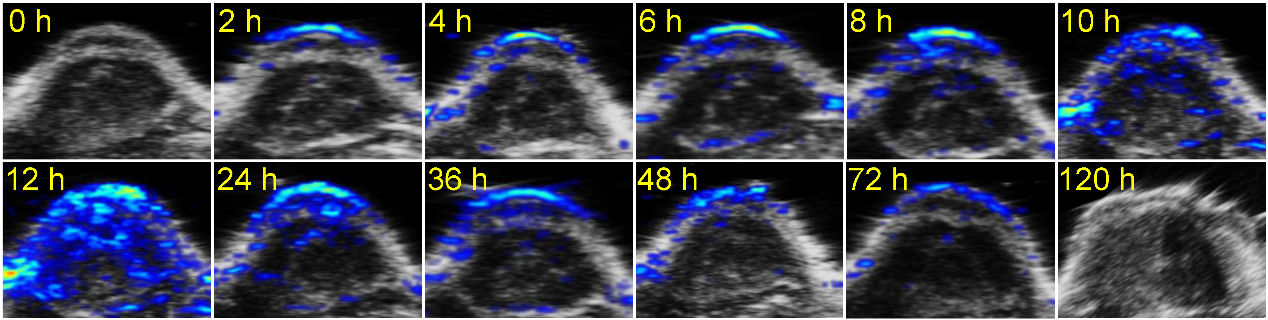


**Figure S6.** Photoacoustic (PA) images of mice after intravenous injection of HPCOF at different time points (0 h, 2 h, 4 h, 6 h, 8 h, 10 h, 12 h, 24 h, 36 h, 2 days, 3 days, and 5 days).


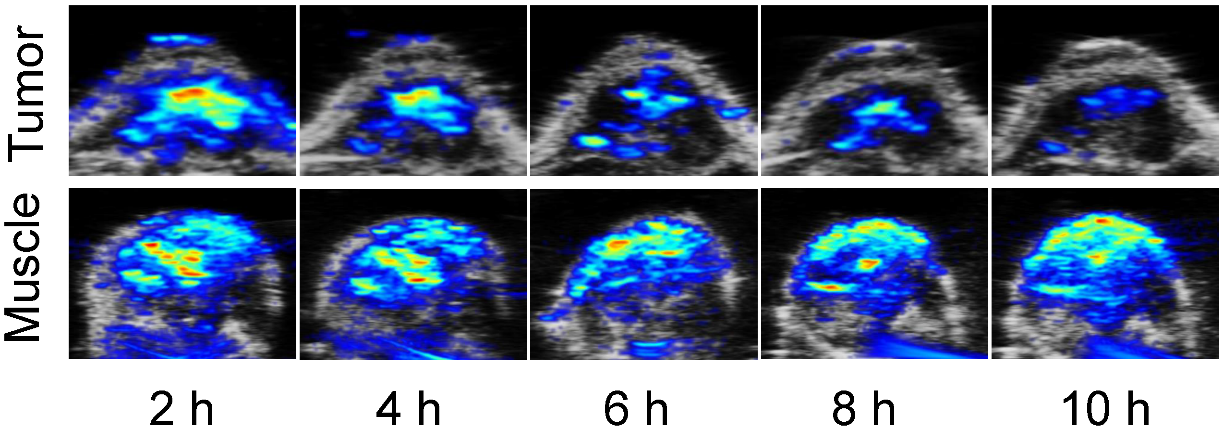


**Figure S7.** PA images of muscle or tumor at different points after intravenous injection of HPCOF.


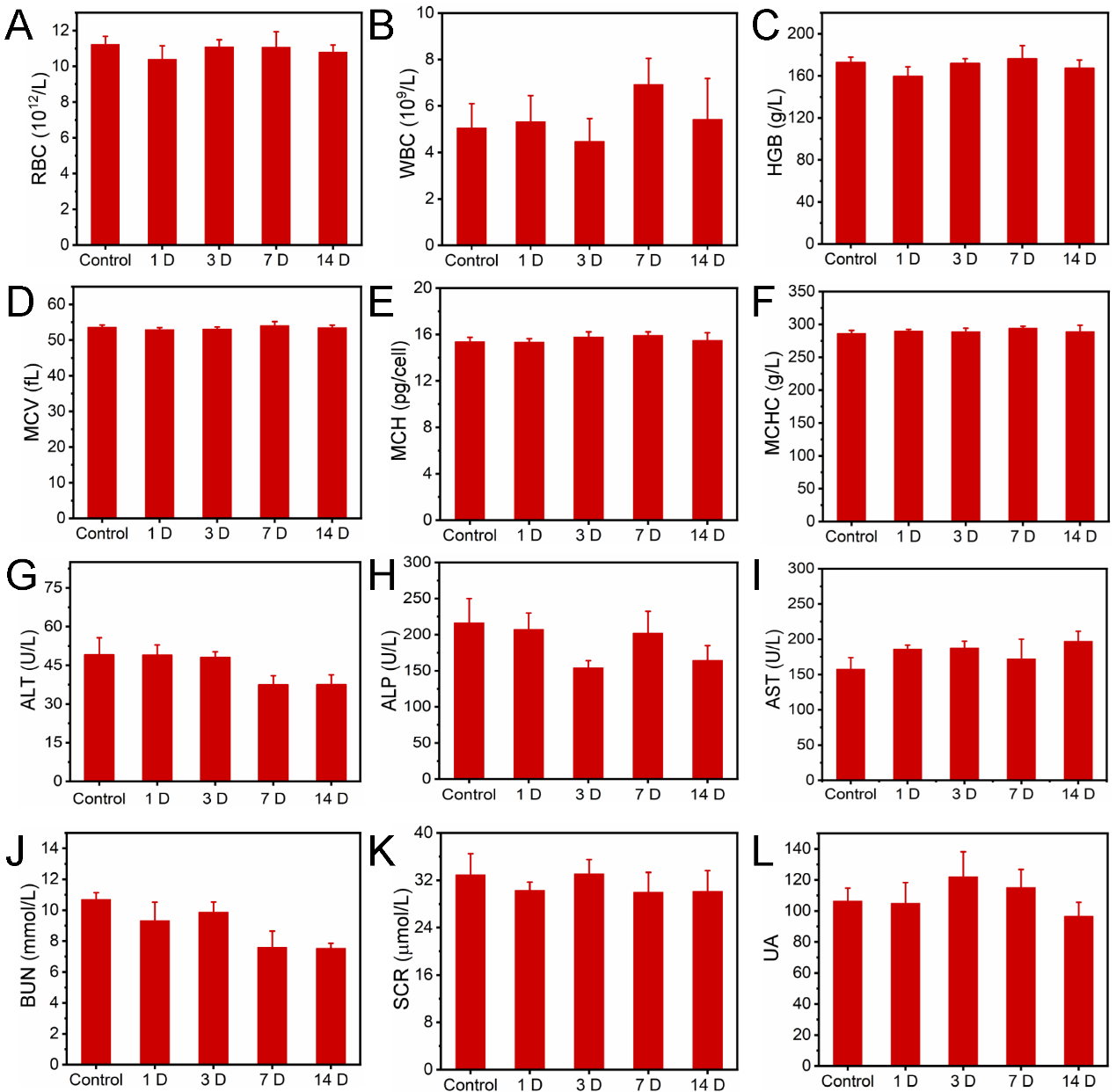


**Figure S8.** Routine blood tests and blood biochemistry of healthy mice after intravenous injection of HPCOF at a dose of 10 mg kg^-1^ for 1 day, 3 days, 7 days, and 14 days. Healthy mice injected with PBS were taken as control. A-F) blood routine indexes of A) red blood cells (RBC), B) while blood cells (WBC), C) haemoglobin (HGB), D) mean corpuscular volume (MCV), E) mean corpuscular haemoglobin (MCH), H) mean corpuscular haemoglobin concentration (MCHC). G-I) Liver function indexes of G) alanine aminotransferase (ALT), H) alkaline phosphatase (ALP), I) asparagine aminotransferase (AST). J-L) Renal function indexes of J) blood urea nitrogen (BUN), K), serum creatinine (SCR), L) uric acid (UA).


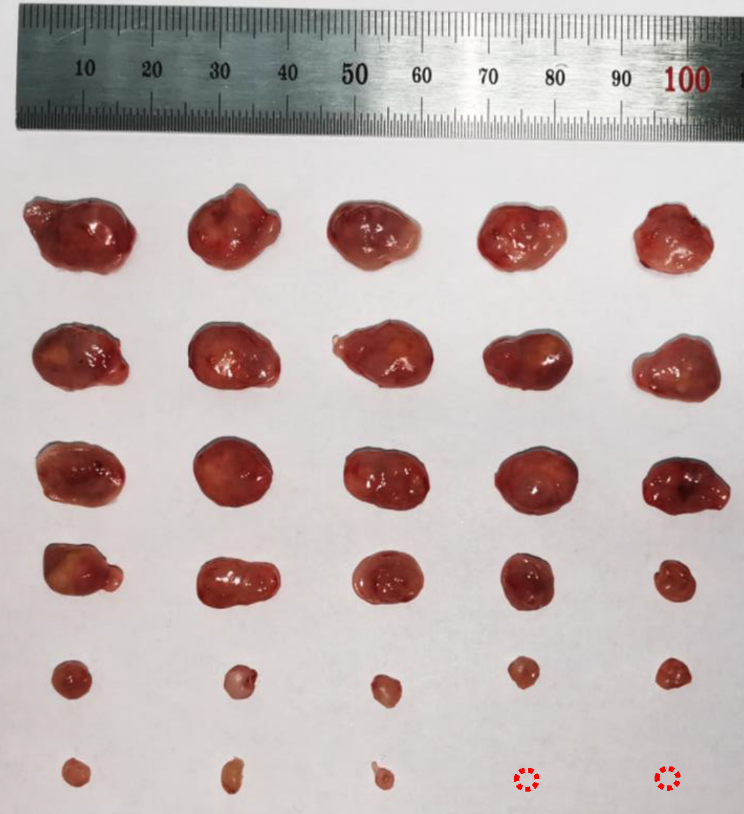


**Figure S9.** Photographs of tumors harvested from tumor-bearing mice in different groups after treatment.
